# Evidence-based practice within nutrition: what are the barriers for improving the evidence and how can they be dealt with?

**DOI:** 10.1186/s13063-017-2160-8

**Published:** 2017-09-11

**Authors:** Martine Laville, Berenice Segrestin, Maud Alligier, Cristina Ruano-Rodríguez, Lluis Serra-Majem, Michael Hiesmayr, Annemie Schols, Carlo La Vecchia, Yves Boirie, Ana Rath, Edmund A. M. Neugebauer, Silvio Garattini, Vittorio Bertele, Christine Kubiak, Jacques Demotes-Mainard, Janus C. Jakobsen, Snezana Djurisic, Christian Gluud

**Affiliations:** 1 Centre de Recherche en Nutrition Humaine Rhone-Alpes, French Obesity Centre of Excellence (FCRIN-FORCE), Lyon 1 University, Hospices Civils de Lyon, Lyon, France; 2 Nutrition Research Group, Research Institute of Biomedical and Health Sciences, University of Las Palmas de Gran Canaria, Las Palmas, Spain; 3 Ciber Fisiopatología Obesidad y Nutrición (CIBEROBN, CB06/03), Instituto de Salud Carlos III (ISCII), Spanish Goverment, Madrid, Spain; 4 Division of Cardiac, Thoracic, Vascular Anaesthesia and Intensive Care, Vienna General Hospital, Währinger Gürtel, Vienna, Austria; 5 Department of Respiratory Medicine, NUTRIM School of Nutrition and Translational Research in Metabolism, Maastricht University Medical Centre, Maastricht, The Netherlands; 6 Department of Clinical Sciences and Community Health, Università degli Studi di Milano Via Vanzetti, Milan, Italy; 7 Service de Nutrition Clinique, CHU de Clermont-Ferrand, Unité de Nutrition Humaine, Clermont-Ferrand, France; 8 Orphanet, Institut National de la Santé et de la Recherche Médicale (INSERM), Paris, France; 9 Brandenburg Medical School, Neuruppin, and Witten/Herdecke University, Witten, Germany; 10 IRCCS Istituto di Ricerche Farmacologiche Mario Negri, Milan, Italy; 11 European Clinical Research Infrastructure Network (ECRIN), Paris, France; 12 Copenhagen Trial Unit, Centre for Clinical Intervention Research, Department 7812, Rigshospitalet, Copenhagen University Hospital, Blegdamsvej 9, DK 2100 Copenhagen, Denmark; 13 Department of Cardiology, Holbæk Hospital, Holbaek, Denmark

**Keywords:** Randomised clinical trials, Evidence-based clinical practice, Evidence-based medicine, Assessment, Specific barriers, Nutrition, ECRIN, European Clinical Infrastructure Network

## Abstract

**Background:**

Evidence-based clinical research poses special barriers in the field of nutrition. The present review summarises the main barriers to research in the field of nutrition that are not common to all randomised clinical trials or trials on rare diseases and highlights opportunities for improvements.

**Methods:**

Systematic academic literature searches and internal European Clinical Research Infrastructure Network (ECRIN) communications during face-to-face meetings and telephone conferences from 2013 to 2017 within the context of the ECRIN Integrating Activity (ECRIN-IA) project.

**Results:**

Many nutrients occur in multiple forms that differ in biological activity, and several factors can alter their bioavailability which raises barriers to their assessment. These include specific difficulties with blinding procedures, with assessments of dietary intake, and with selecting appropriate outcomes as patient-centred outcomes may occur decennia into the future. The methodologies and regulations for drug trials are, however, applicable to nutrition trials.

**Conclusions:**

Research on clinical nutrition should start by collecting clinical data systematically in databases and registries. Measurable patient-centred outcomes and appropriate study designs are needed. International cooperation and multistakeholder engagement are key for success.

**Electronic supplementary material:**

The online version of this article (doi:10.1186/s13063-017-2160-8) contains supplementary material, which is available to authorized users.

## Background

The European Clinical Research Infrastructure Network Integrating Activity (ECRIN-IA) project has initiated work to identify threats and barriers to evidence-based clinical practice [1–3].^1^

We identified common barriers affecting all clinical trials, namely inadequate knowledge and understanding of clinical research and trial methodology; lack of funding; excessive monitoring; restrictive interpretation of privacy law and lack of transparency; overly complex or inadequate regulatory requirements; and inadequate clinical research infrastructures [4]. Furthermore, we identified barriers towards the conduct of clinical research on rare diseases, including the direct consequence of rarity; limited knowledge on the natural history of rare diseases; the need for trial designs adapted to the small population size and clinical heterogeneity; the need for multinational randomised clinical trials and infrastructures to conduct such trials; the need for more sensitive outcomes to quantify clinical benefit; and the need for involvement of all the stakeholders in the study design and conduct [5]. In a related paper, we also assess the specific barriers towards clinical research on medical devices [6]. In the present paper, we assess the specific barriers towards clinical research on nutrition and examine how they can be broken down in order to improve the future evidence base.

Due to the ubiquitous nature of nutrition and the multiple metabolic effects induced by each food, nutrient, or micronutrient component, randomised clinical trials (RCTs) in this area face practical barriers [7]. By nature, some barriers can be difficult to resolve such as making an appropriate placebo for blinding of the interventions. Due to the multiple metabolic impact of nutrients, choosing the primary outcome and the determination of sample size may seem particularly difficult. Nutrition research on products is also complex because it exploits a multitude of bioactive compounds acting on an extensive network of interacting processes. In addition to measuring the variables of interest (assessing health), an extensive description of the trial participants and foods or diets consumed is essential. However, solutions exist to respect general rules of clinical trials despite these barriers.

The present paper gives an overview of the specific obstacles in methodology within nutrition research and provides solutions to these barriers (Table 1). The regulations in this area are also discussed.

**Table 1:** Barriers to the conduct of randomised clinical trials (RCTs) on nutrition

Specific barriers to RCTs on nutrition	Comments
Testing a nutrient	Should one use the nutrient as an add-on to usual diet, or should the diet be depleted of the nutrient?
Testing a food intervention	How to assess dietary intake
Assessing dietary intake	Combination of diverse methods including the 24-h dietary recall method are likely to be best
Selection of outcomes	Depends on the objective of the study

Barriers as identified by the European Clinical Research Infrastructure Network (ECRIN) panel in the context of the ECRIN-IA project. These barriers are additional to the barriers that affect all clinical trials and trials on rare diseases [4, 5]

## Methods

**Fig. 1:**
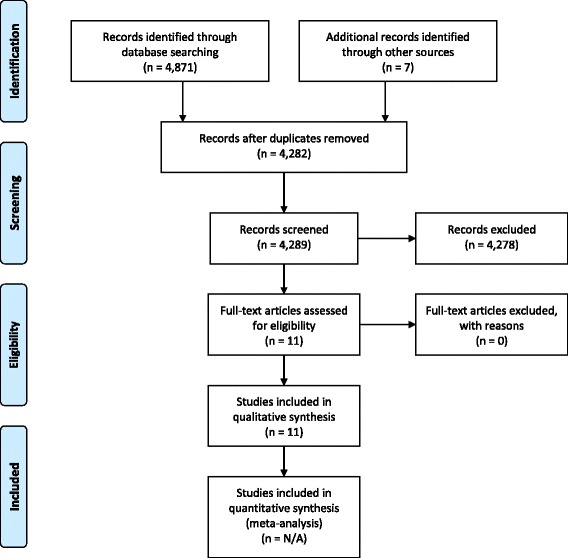
Preferred Reporting Items for Systematic Reviews and Meta-analyses (PRISMA) 2009 flow diagram. PRISMA flow diagram depicting the selection process of relevant academic literature

The present study is based on a combination of systematic academic literature searches as well as internal ECRIN-IA communications from 2013 to 2017. In May 2016, the systematic search was performed using The Cochrane Library (Wiley) (Issue 5 of 12, 2016) (including the Cochrane Database of Systematic Reviews (CDSR), CENTRAL, National Health Service Economic Evaluation Database (NHSEED), and Database of Abstracts of Reviews of Effects (DARE, U.S. Library of Medicine)); MEDLINE (Ovid SP) (1946 to May 2016); EMBASE (Ovid SP) (1974 to May 2016); and Science Citation Index Expanded (1900 to May 2016) using different terms covering barriers, evidence-based medicine, and nutrition. The exact search strategy is provided in Additional file 1. A Preferred Reporting Items for Systematic Reviews and Meta-analyses (PRISMA) flow diagram depicting the selection process and a PRISMA Checklist are provided in Fig. 1 and Additional file 2, respectively. Articles were selected if they included valid considerations on how barriers to the conduct of randomised clinical trials (RCTs) on nutrition could affect their number, feasibility, and quality.

## Results and discussion

### Search results

Through the electronic searches a total of 4871 references were identified. After 589 duplicates were removed, a total of 4282 references remained, and were further reduced to yield 11 relevant references (Additional file 3).

### Randomised clinical trials on nutrition

RCTs are costly and labourious to conduct, but they are essential [8–11]. The RCT is the only design which is able to demonstrate a causal relationship between a dietary change and health outcomes [8–11]. However, testing the effect of a diet or a specific nutrient in a trial is not the same as testing a drug and different obstacles are encountered (Table 1).

#### Testing a nutrient

If only a nutrient is tested, a placebo can be easily used, and an RCT with blinding can be conducted. Compliance can be checked by counting pills and possibly by assaying the nutrient concentration or related functions.

However, many nutrients occur in multiple forms that differ in biological activity, and there are several factors that can alter the bioavailability of individual nutrients. The nutrient status and the baseline exposure could also cause interference. The nutrient status of an individual or a population can affect the response to nutrient supplementation, e.g., via the gastrointestinal microbiota. For example, if one wants to examine the effect of polyphenol, adding polyphenol to polyphenol-depleted diets will give a good check on polyphenol dose. However, these special depleted diets (depleted in fruit, vegetable, and polyphenol-containing beverages) will have confounding effects and there will be no real control group. Another way will be to add polyphenol in addition to the polyphenols of a normal diet, but then both groups might receive sufficient polyphenol, and a possible effect of polyphenol might be overlooked. Both types of trials will give information, but different information. Background levels of exposures can be difficult to accurately determine due to limitations in currently available assessment methodologies of food intake, incomplete nutrient databases with which nutrient intake estimates are calculated, and temporal changes in exposure. This must be carefully considered in the trial design, including randomisation and subgroup analysis per baseline status. For smaller RCTs (say less than 400 to 500 participants), adjustment according to the baseline level of consumption or nutrient level in blood may be necessary [12].

#### Testing a food intervention

Food-based interventions are complex; an appropriate placebo for food cannot be designed so RCTs cannot easily be blinded. However, outcome assessors, data collectors, statisticians, and conclusion drawers should be blinded. Furthermore, a specific design with food made with or without the particular nutrient can be conducted.

Thus, food-based nutrition interventions, in contrast to nutrient supplementation, present unique challenges in accurately quantifying the absolute change in intake. Compliance assessment in these trials is more difficult but biomarkers of food intake may be used (alpha-linolenic acid as a proxy for nut intake or some polyphenols, such as urinary tyrosol, for virgin olive oil) [13]. Certain periods of holidays or religious festivities (such as Ramadan) should be avoided to reduce interference from special nutritional patterns during such periods. The seasonal impact on food intake also needs to be taken into account [14]. The optimal length of intervention to have a relevant effect can also be difficult to determine. Here again, particular attention must be paid when testing a food product, the product will not have the same impact if it substitutes another product or if it is added to the usual diet.

The ILSI Europe Functional Foods Task Force guidelines [15] are a relevant source on how to conduct nutrition intervention studies and can help resolve some of those questions.

#### Assessing dietary intake

Assessing dietary intake is a major challenge in nutrition research despite the large spectrum of research methodologies available.

Quantification of habitual dietary exposure by measurement of food intake combined with food composition is essential for investigating the impact of food on health. The assessment of dietary intake at the population level provides important information on the frequency and distribution of inadequate diets and nutritional status and guides the design of population-based interventions targeting the improvement of dietary habits at the community level. Obtaining reliable data on food consumption (identifying the intake of energy and nutrients) is a key factor and a necessary tool in health promotion and prediction of disease risk [16].

Difficulties are encountered in the assessment of dietary intake. Valid methods are required to assess the effects of a huge range of diverse foods and compounds (macro- and micronutrients, and non-nutritional bioactive compounds) on individual health. A wide variety of dietary survey methods exists, with each one presenting a series of advantages and disadvantages that must be taken into consideration based on the study aims, the degree of precision needed and the available resources. Common methods involving dietary questionnaires are:

- Food frequency
- 24-h recall
- Dietary history over a specific period
- Food diary associated with photographic food record
- Weighed food record
- Specific dietary items or patterns

The validity of these approaches has been questioned many times [17]. A volunteer assessing their dietary intake may modify their eating behaviour, thus introducing an another issue in dietary assessment. Dietary intake is difficult to measure and a single instrument that is optimal for all settings does not exist, as each method has its pros and cons or practical difficulties that should be taken into account when selecting the instrument to be administered. For example, a weighed-food record is precise but rather cumbersome for the volunteers and can lead to underreporting. The method used could be improved by using more frequent dietary recall.

Expectations for better assessment of dietary intake are put on new methods, such as research in metabolomics, metagenomics, and natural enrichment of stable isotopes, which could be helpful in terms of the validation of methods and useful in addition to standard methods. Technological devices, such as cellular phones, may also improve the quality of data collected. The majority of the latest-generation technologies offer interesting tools for the process of evaluating dietary intake in epidemiological studies [18–21]. The advantages of digital instruments include: (1) reduction in interviewer bias; (2) reduction in the time and cost of field work; (3) data collection and codification in real time; (4) automatic calculation of daily intake; and (5) highly economic options of capturing food intake online (computer, tablet, and smartphone).

New technologies provide many possibilities for assessing dietary intake in individuals and groups, but are not free of certain limitations: high cost of programme design and acquisition of devices; difficulty of application to certain populations that are not familiar with new technologies; and dependence on access to Internet and on the trial participant’s recall capacity.

Despite the interesting progress and the incorporation of innovative technology into dietary assessment methods, the same flaws remain. As such, the combination of diverse methods could be the best approach, with the 24-h dietary recall being a rather reliable comprehensive and easy-to-use instrument [22–24].

Research over recent years in this field has focussed on estimating the adherence to a determined dietary pattern rather than analysing the individual components of the diet in relation to the health of the population. Many indirect indices have been proposed. These indices summarise the diet by means of a single score that results from a function of numerous components (previously selected on the basis of prior knowledge or scientific evidence) such as food, food groups, or a combination of foods and nutrients [25]. The validity of these indices has been evaluated by examining their relationship with nutrient adequacy and several health outcomes (i.e., nutrient-related diseases). For instance, indices evaluating adherence to the Mediterranean diet, like the Mediterranean food pattern PREDIMED study (MeDiet-PREDIMED) [26], the Mediterranean Adequacy Index (MAI) [27], the Mediterranean Diet Score (MDS) [28], or the Mediterranean Diet Quality Index (KIDMED) for evaluating the quality of the Mediterranean diet in children and adolescents [29], have been successfully applied to studies on life expectancy [30], cardiovascular risk [31–33], cancer [34, 35], hypertension [36], obesity [37, 38], and diabetes [39]. The study of dietary patterns using dietary quality indices to examine interactions between dietary components and their effect on several health variables can be complementary to the analysis of single dietary components [40, 41]. Moreover, these indices are also useful tools to measure food consumption trends and to identify the involved factors, as well as to develop comprehensive public health nutrition recommendations.

Approaches to evidence-based medicine, with its reliance on RCTs and systematic reviews of RCTs [1] have been adapted to nutrition systematic reviews and policy [42–45]. However, there are distinct differences between the evidence that can be obtained for the testing of drugs using RCTs and those needed for the development of nutrient requirements or dietary guidelines [46]. Nutrients tend to work in systems in concert with other nutrients and affect multiple cells and organs; nutrients are homeostatically controlled and thus the body’s baseline nutrient ‘status’ affects the response to a nutrient intervention; a nutrient intervention group cannot be contrasted with a true placebo group (i.e., a ‘zero-exposure’ group). The subtle effect of nutrients and small effect sizes means that far more participants are needed to demonstrate statistical significance depending on the research question and the target population.

Chronic diseases can take decades to develop, so demonstrating a statistically significant and clinically relevant reduction in risk with any intervention requires very long-term trials. Moreover, maintaining adherence to a dietary change all through a trial period is difficult. RCTs present one approach toward understanding the efficacy of nutrient interventions, but the innate complexities of nutrient actions and interactions cannot always be adequately addressed through a single research design.

#### Outcomes

Outcomes could vary according to the objectives of a study. In mechanistic proof-of-concept studies, they could be very specific and explanatory [47]. The sample size should be calculated according to the variability of the measured primary outcome which could be either a single outcome or a composite outcome.

Secondary outcomes should have enough power (at least 80%) to determine a conclusion; if not, they should only be used as exploratory outcomes [1]. Outcomes should concern nutritional status, relevant markers of functional status, quality of life, morbidity, and mortality. Hard outcomes should always be preferred when assessing the effects of clinical nutrition interventions, whereas intermediate markers might be used in addition to hard outcomes in preventive nutrition.

Numerous experimental nutrition studies have used putative surrogate outcomes and markers of disease risk, but only a few such markers have been validated using hard outcomes [1]. Nutrition research needs better biomarkers of both exposures and outcome. Defining approved panels of outcomes for particular health outcomes would allow easier conduct of systematic reviews and meta-analyses. It is mandatory to have precise outcomes to predefine the moment of their measurement, as is the case for all types of research, and to decide them before commencing RCTs [1].

For dietary supplements and functional food, studies could also target non-diseased populations. Statistically, however, there are challenges in performing studies in a non-diseased, healthy population. In healthy volunteers, the magnitude of functional changes and their clinical relevance are likely to be more difficult to highlight than it would be in diseased participants and the likelihood of demonstrating a statistically significant difference is diminished. Experimental designs including metabolic challenges (such as short-term overfeeding or a fat or fructose load) could be useful to screen for the protective effect of some nutrients. Studies in high-risk populations are encouraged.

The need to accurately capture subtle changes in a multitude of variables creates several challenges. Standardised technology, methodology, and data formats are required for meeting these challenges. Elements of these issues are common to all biological sciences and efforts to produce solutions and best practices for technologies and data handling in these areas are under investigation [48].

Single-centre trials are easier to conduct than multicentre trials and may be done more efficiently with a homogenous population. However, they have less external validity and often do not reach a required sample size. Accordingly, it is often necessary to organise multicentre trials in order to achieve an adequate sample size. In general, results from a multicentre trial will be more convincing than those coming from a single centre.

Solutions to the specific problems of nutrition research could be solved by the extension of good clinical research practice and harmonising procedures.

#### System studies and observational studies

Observational studies derived from large epidemiological surveys are able to raise hypotheses in terms of the association between dietary patterns and health outcomes [49].

These hypotheses, as well as other hypothesis derived from experimental research, should be the basis for RCTs.

More and more observational and epidemiological studies, which are carried out on a dietary basis, have attempted to associate the intake of particular food group with disease prevalence. The results are rarely unidirectional, and it still remains unclear whether certain food groups may be considered as definitively protective or deleterious. Therefore, a more holistic approach based on cumulative scientific evidence, i.e., data mining, may be needed [50]. This global approach draws updated pictures on the research led on such associations and helps to unravel research needs. For instance, based on meta-analyses and systematic reviews of articles from 1950 to 2013 referring to observational and intervention studies, we were able to identify tendencies related to the health-protectiveness of 17 food groups and beverages towards 10 disease risks [51]. Among these tendencies, some can be considered as strong and may potentially be converted into clear and durable recommendations for public policies related to preventive nutrition and health, while others remain ambiguous.

Another important aspect of nutrition research is that exposure to specific food groups or nutrients may induce metabolic disorders long before any non-communicable chronic disease may be detected in relation to the diet. The origins of these diseases are multifactorial and may result from at least 10 different deregulated metabolic variables, including antioxidant status, acid-base imbalance, increased inflammatory status, impaired carbohydrate/lipid/one-carbon metabolism, impaired functioning of neurons and deoxyribonucleic acid (DNA) transcription, hypertension and/or modified digestive microbiota [52]. Such a systems approach represents an important opportunity to identify early biomarkers of future chronic diseases in association with dietary nutrients. So, technological development allowing rapid and large screening of metabolites, i.e., metabolomics, in plasma or urine samples, will likely be of high value for detecting dietary risk factors in individuals [53].

#### Regulations

The level of the quality of nutrition research is also dependent on regulations and practices that have been approved by EFSA (The European Food Safety Authority). The authority has indicated a preference for clinical studies to back health claims [13]. Placebo-controlled trials are the ‘gold standard’ with either a parallel or crossover design. RCTs involving whole foods or diets are difficult to conduct, perhaps even more so than nutrient-based trials, but for the same reasons: lack of compliance, no true placebo group, and the impossibility of having a control group. Furthermore, the innate complexities of nutrient actions and interactions cannot always be adequately addressed through any single research design.

Good clinical practice is applicable to interventional research beyond health products, particularly in nutrition, to ensure the reliability and reproducibility of the data and to safeguard the rights and safety of research participants and the confidentiality of information that concern them [54, 55].

The great heterogeneity of national regulations regarding non-drug research mainly depends on the consideration of a dietary supplement as a drug or not and the level of intervention imposed on the patient. There is a need to harmonise the procedures for evaluating nutrition clinical trials in Europe. One of the deliverables of WP6 within ECRIN-IA has been to collect the different national regulation requirements and develop a pan-European human nutrition regulatory and ethics database [56].

For the US Food and Drug Administration (FDA), if an Investigational New Drug (IND) is needed for a clinical investigation, evaluating a dietary supplement is determined by the intent of the clinical investigation. If the clinical investigation is intended only to evaluate the dietary supplement’s effect on the structure or function of the body, an IND is not required. However, if the clinical investigation is intended to evaluate the dietary supplement’s ability to diagnose, cure, mitigate, treat, or prevent a disease, an IND is required under part 312 [54]. The European Commission has now adopted a revision of the Directive on Clinical Trials (2001-20-EC) 17 July 2012 [57], Regulation EU No. 536/2014 which will come into force in 2019 at the earliest [58]. This should facilitate the conduct of multinational clinical trials with particular attention to the acceleration and simplification of licensing and reporting clinical trials procedures. Through this proposed regulation, it will be possible to ensure, in all member states, identical rules for the authorisation, execution, and monitoring of clinical trials.

## Conclusions

Nutrition research must apply reproducible and valid methodologies [8, 15]. The methodologies and regulations for drug trials are applicable to trials in the field of nutrition. A consensual approach involving institutional and industrial partners of the clinical nutrition research would be useful for standardisation of these practices on specific aspects such as the choice of a placebo, the design of the test products, the selection of control participants, the choice of outcomes, and sample size estimation. In collaboration with ECRIN, it will be necessary to standardise nutritional and outcome assessment, standardise operating procedures for existing and new methodologies, and standardise data collection, data management and data sharing, which will make studies comparable and interoperable for systematic reviews meta-analyses.

Taking into account the specificities of nutrition studies and developing international collaboration will enhance the quality of nutrition research [1].

## Additional files

Additional file 1: Literature search strategy. Description of data: exact search strategy applied for analyses. (DOCX 14 kb)

Additional file 2: PRISMA 2009 Checklist. (DOC 63 kb)

Additional file 3: Relevant references from the academic literature search. Results from the academic literature search are listed in the form of relevant publications. (DOCX 14 kb)

## Abbreviations

CDSR: Cochrane Database of Systematic Reviews
ECRIN: European Clinical Research Infrastructure Network
ECRIN-IA: European Clinical Research Infrastructure Network Integrating Activity
EFSA: European Food Safety Authority
FDA: US Food and Drug Administration
IND: Investigational New Drug
KIDMED: Mediterranean Diet Quality Index
MAI: Mediterranean Adequacy Index
MDS: Mediterranean Diet Score
MeDiet-PREDIMED: Mediterranean food pattern PREDIMED study
NHSEED: National Health Service Economic Evaluation Database
RCT: Randomised clinical trial

## Footnotes

1 Funded by the European Union Framework Programme 7 (EU FP7; grant agreement no. 284395), ECRIN-IA involved 23 countries and brought together diverse stakeholders to overcome barriers to clinical research in three particularly difficult areas (rare diseases, medical devices, and nutrition). Specifically, the project aimed to develop tools, services, and infrastructure to facilitate multinational clinical research in Europe, and to support the development of pan-European disease networks to drive clinical projects. This in turn was intended to improve Europe’s attractiveness to industry, boost its scientific competitiveness, and result in better health care for European citizens. Originally planned for 4 years (2012 to 2015), the clinical trials work package was extended until 2017.

## References

[1] Garattini S, Jakobsen JC, Wetterslev J, Bertele V, Banzi R, Rath A (2016). Evidence-based clinical practice: overview of threats to the validity of evidence and how to minimise them. Eur J Intern Med.

[2] European Clinical Research Infrastructure Network. Capacity building projects. European Clinical Research Infrastructure Network Integrating Activity (ECRIN-IA). 2016. Available from: http://www.ecrin.org/activities/projects. Accessed on 16 Dec 2016.

[3] Demotes-Mainard J, Kubiak C. A European perspective—the European Clinical Research Infrastructures Network. Ann Oncol. 2011;22(Suppl 7):vii 44–9. Epub 9 Nov 2011. 10.1093/annonc/mdr425 22039144

[4] Djurisic S, Rath A, Gaber S, Garattini S, Bertele V, Ngwabyt SN (2017). Barriers to the conduct of randomised clinical trials within all disease areas. Trials.

[5] Rath A, Salamon V, Peixoto S, Hivert V, Laville M, Masson Y (2017). A systematic literature review of evidence-based clinical practice for rare diseases: what are the perceived and real barriers for improving the evidence and how can they be overcome? Trials [accepted for publication].

[6] Neugebauer EAM, Rath A, Antoine S-L, Eikermann M, Seidel D, Koenen C, et al. Specific barriers to the conduct of randomised clinical trials on medical devices. Trials [accepted for publication]. 2017. 10.1186/s13063-017-2168-0 PMC5597993 28903769

[7] Meunier N, Roth H, Ferrand L, Laville M, Cano N (2010). La recherche clinique en nutrition—Méthodologie et réglementation des essais cliniques. Nutr Clin Metab.

[8] Ioannidis JP (2016). We need more randomized trials in nutrition—preferably large, long-term, and with negative results. Am J Clin Nutr.

[9] Jakobsen JC, Gluud C (2013). The necessity of randomized clinical trials. Br J Med Res.

[10] Maki KC, Slavin JL, Rains TM, Kris-Etherton PM (2014). Limitations of observational evidence: implications for evidence-based dietary recommendations. Adv Nutr.

[11] Ioannidis JP (2013). Implausible results in human nutrition research. BMJ.

[12] Kernan WN, Viscoli CM, Makuch RW, Brass LM, Horwitz RI (1999). Stratified randomization for clinical trials. J Clin Epidemiol.

[13] Estruch R, Ros E, Salas-Salvado J, Covas MI, Corella D, Aros F (2013). Primary prevention of cardiovascular disease with a Mediterranean diet. N Engl J Med.

[14] Dominguez-Salas P, Moore SE, Cole D, da Costa KA, Cox SE, Dyer RA (2013). DNA methylation potential: dietary intake and blood concentrations of one-carbon metabolites and cofactors in rural African women. Am J Clin Nutr.

[15] Welch RW, Antoine JM, Berta JL, Bub A, de Vries J, Guarner F, Hasselwander O, Hendriks H, Jäkel M, Koletzko BV, Patterson CC, Richelle M, Skarp M, Theis S, Vidry S, Woodside JV (2011). International Life Sciences Institute Europe Functional Foods Task Force guidelines for the design, conduct and reporting of human intervention studies to evaluate the health benefits of foods. Br J Nutr..

[16] Salvador Castell G, Serra-Majem L, Ribas-Barba L (2015). What and how much do we eat? 24-hour dietary recall method. Nutr Hosp.

[17] Poslusna K, Ruprich J, de Vries JH, Jakubikova M, van’t Veer P (2009). Misreporting of energy and micronutrient intake estimated by food records and 24 hour recalls, control and adjustment methods in practice. Br J Nutr.

[18] Shim JS, Oh K, Kim HC (2014). Dietary assessment methods in epidemiologic studies. Epidemiol Health.

[19] Illner AK, Freisling H, Boeing H, Huybrechts I, Crispim SP, Slimani N (2012). Review and evaluation of innovative technologies for measuring diet in nutritional epidemiology. Int J Epidemiol.

[20] Shriver BJ, Roman-Shriver CR, Long JD (2010). Technology-based methods of dietary assessment: recent developments and considerations for clinical practice. Curr Opin Clin Nutr Metab Care.

[21] Hercberg S (2012). Web-based studies: the future in nutritional epidemiology (and overarching epidemiology) for the benefit of public health?. Prev Med.

[22] Majem LS, Bartrina JA (2006). Nutricion y salud publica. Metodos, bases cientıficas y aplicaciones (Public Health Nutrition. Evidence base and applications).

[23] Martin-Moreno JM, Gorgojo L (2007). Valoración de la ingesta dietética a nivel poblacional mediante cuestionarios individuales: sombras y luces metodológicas. Rev Esp Salud Publica.

[24] Hercberg S, Deheeger M, Preziosi P (1994). SU-VI-MAX. Portions alimentaires. Manuel photos pour l’estimation des quantités.

[25] Bach A, Serra-Majem L, Carrasco JL, Roman B, Ngo J, Bertomeu I (2006). The use of indexes evaluating the adherence to the Mediterranean diet in epidemiological studies: a review. Public Health Nutr.

[26] Sanchez-Tainta A, Estruch R, Bullo M, Corella D, Gomez-Gracia E, Fiol M (2008). Adherence to a Mediterranean-type diet and reduced prevalence of clustered cardiovascular risk factors in a cohort of 3,204 high-risk patients. Eur J Cardiovasc Prev Rehabil.

[27] Alberti-Fidanza A, Fidanza F, Chiuchiu MP, Verducci G, Fruttini D (1999). Dietary studies on two rural Italian population groups of the Seven Countries Study. 3. Trend of food and nutrient intake from 1960 to 1991. Eur J Clin Nutr.

[28] Trichopoulou A, Orfanos P, Norat T, Bueno-de-Mesquita B, Ocke MC, Peeters PH (2005). Modified Mediterranean diet and survival: EPIC-elderly prospective cohort study. BMJ.

[29] Serra-Majem L, Ribas L, Ngo J, Ortega RM, Garcia A, Perez-Rodrigo C (2004). Food, youth and the Mediterranean diet in Spain. Development of KIDMED, Mediterranean Diet Quality Index in children and adolescents. Public Health Nutr.

[30] Trichopoulou A, Kouris-Blazos A, Wahlqvist ML, Gnardellis C, Lagiou P, Polychronopoulos E (1995). Diet and overall survival in elderly people. BMJ.

[31] Martinez-Gonzalez MA, Garcia-Lopez M, Bes-Rastrollo M, Toledo E, Martinez-Lapiscina EH, Delgado-Rodriguez M (2011). Mediterranean diet and the incidence of cardiovascular disease: a Spanish cohort. Nutr Metab Cardiovasc Dis.

[32] Mar Bibiloni M, Martinez E, Llull R, Maffiotte E, Riesco M, Llompart I (2011). Metabolic syndrome in adolescents in the Balearic Islands, a Mediterranean region. Nutr Metab Cardiovasc Dis.

[33] Martinez-Gonzalez MA, Fernandez-Jarne E, Serrano-Martinez M, Marti A, Martinez JA, Martin-Moreno JM (2002). Mediterranean diet and reduction in the risk of a first acute myocardial infarction: an operational healthy dietary score. Eur J Nutr.

[34] Buckland G, Agudo A, Lujan L, Jakszyn P, Bueno-de-Mesquita HB, Palli D (2010). Adherence to a Mediterranean diet and risk of gastric adenocarcinoma within the European Prospective Investigation into Cancer and Nutrition (EPIC) cohort study. Am J Clin Nutr.

[35] La Vecchia C, Bosetti C (2006). Diet and cancer risk in Mediterranean countries: open issues. Public Health Nutr.

[36] Toledo E, de A Carmona-Torre F, Alonso A, Puchau B, Zulet MA, Martinez JA (2010). Hypothesis-oriented food patterns and incidence of hypertension: 6-year follow-up of the SUN (Seguimiento Universidad de Navarra) prospective cohort. Public Health Nutr.

[37] Romaguera D, Norat T, Mouw T, May AM, Bamia C, Slimani N (2009). Adherence to the Mediterranean diet is associated with lower abdominal adiposity in European men and women. J Nutr.

[38] Yannakoulia M, Panagiotakos D, Pitsavos C, Lentzas Y, Chrysohoou C, Skoumas I (2009). Five-year incidence of obesity and its determinants: the ATTICA study. Public Health Nutr.

[39] Martinez-Gonzalez MA, de la Fuente-Arrillaga C, Nunez-Cordoba JM, Basterra-Gortari FJ, Beunza JJ, Vazquez Z (2008). Adherence to Mediterranean diet and risk of developing diabetes: prospective cohort study. BMJ.

[40] Brown TA (2006). Confirmatory factor analysis for applied research.

[41] Hu FB (2002). Dietary pattern analysis: a new direction in nutritional epidemiology. Curr Opin Lipidol.

[42] Bjelakovic G, Nikolova D, Gluud LL, Simonetti RG, Gluud C (2012). Antioxidant supplements for prevention of mortality in healthy participants and patients with various diseases. Cochrane Database Syst Rev.

[43] Feinberg J, Nielsen E, Korang S, Engell K, Rasmussen M, Zhang K (2016). OR29: nutrition support in hospitalised adults at nutritional risk. A Cochrane systematic review with meta-analysis and Trial Sequential Analysis (Abstract). Clin Nutr.

[44] Avenell A, Smith TO, Curtain JP, Mak JC, Myint PK (2016). Nutritional supplementation for hip fracture aftercare in older people. Cochrane Database Syst Rev.

[45] Feinberg J, Nielsen EE, Gluud C, Lindschou J, Kondrup J, Jakobsen JC. Nutrition support in hospitalised adults at nutritional risk (Protocol). Cochrane Database Syst Rev. 2015(Issue 3):CD011598. doi:10.1002/14651858.CD011598. 10.1002/14651858.CD011598.pub2 PMC6481527 28524930

[46] Blumberg J, Heaney RP, Huncharek M, Scholl T, Stampfer M, Vieth R (2010). Evidence-based criteria in the nutritional context. Nutr Rev.

[47] Timmers S, Konings E, Bilet L, Houtkooper RH, van de Weijer T, Goossens GH (2011). Calorie restriction-like effects of 30 days of resveratrol supplementation on energy metabolism and metabolic profile in obese humans. Cell Metab.

[48] van Ommen B, Bouwman J, Dragsted LO, Drevon CA, Elliott R, de Groot P (2010). Challenges of molecular nutrition research 6: the nutritional phenotype database to store, share and evaluate nutritional systems biology studies. Genes Nutr.

[49] Fagherazzi G, Vilier A, Saes Sartorelli D, Lajous M, Balkau B, Clavel-Chapelon F (2013). Consumption of artificially and sugar-sweetened beverages and incident type 2 diabetes in the Etude Epidémiologique auprès des Femmes de la Mutuelle Générale de l’Education Nationale-European Prospective Investigation into Cancer and Nutrition cohort. Am J Clin Nutr.

[50] van Ommen B, Fairweather-Tait S, Freidig A, Kardinaal A, Scalbert A, Wopereis S (2008). A network biology model of micronutrient related health. Br J Nutr.

[51] Fardet A, Boirie Y (2014). Associations between food and beverage groups and major diet-related chronic diseases: an exhaustive review of pooled/meta-analyses and systematic reviews. Nutr Rev.

[52] Fardet A, Boirie Y (2013). Associations between diet-related diseases and impaired physiological mechanisms: a holistic approach based on meta-analyses to identify targets for preventive nutrition. Nutr Rev.

[53] Morio B, Comte B, Martin J-F, Chanseaume E, Alligier M, Junot C (2015). Metabolomics reveals differential metabolic adjustments of normal and overweight subjects during overfeeding. Metabolomics.

[54] Food and Drug Administration. U. S. Department of Health and Human Services. Guidance for industry: oversight of clinical investigations, a risk based approach to monitoring. 2013 [cited 2017 September 06]; Available from: http://www.fda.gov/downloads/Drugs/…/Guidances/UCM269919.pdf.

[55] Regulation (EU) No 536/2014 of the European Parliament and of the Council of 16 April 2014 on clinical trials on medicinal products for human use, and repealing Directive 2001/20/EC. 2014 [cited 2017 September 06]; Available from: http://ec.europa.eu/health/files/eudralex/vol-1/reg_2014_536/reg_2014_536_en.pdf.

[56] European Clinical Research Infrastructures Network—Transnational Working Groups (ECRIN-TWG). Deliverable 5. Meetings on the legislative and regulatory frameworks for clinical research in Europe. 2008 [cited 2017 September 06]; Available from: http://ctu.dk/media/12427/ECRIN_TWG_D5.pdf.

[57] Revision of the EU Clinical Trials Directive—Adoption of the proposal for a ‘Clinical Trials Regulation’—17 July 2012 [cited 2017 September 06]; Available from: http://ec.europa.eu/health/human-use/clinical-trials/index_en.htm.

[58] European Medicines Agency. Clinical trial regulation. 2017. Available from: http://www.ema.europa.eu/ema/index.jsp?curl=pages/regulation/general/general_content_000629.jsp. Accessed on 21 Jul 2017.

