# Supplementary material for: Evidence-based practice within nutrition: what are the barriers for improving the evidence and how can they be dealt with?
Source: Trials. 2017 Sep 11;18:425. doi: 10.1186/s13063-017-2160-8 (PMC5594518; doi:10.1186/s13063-017-2160-8)
Supplement: Supplementary file 3 — Relevant references from the academic literature search. Results from the academic literature search are listed in the form of relevant publications. (DOCX 14 kb) [file 13063_2017_2160_MOESM3_ESM.docx]

Additional file 3. Relevant references from literature search (n=11)

- Brown AW, Ioannidis JP, Cope MB, Bier DM, Allison DB. Unscientific beliefs about scientific topics in nutrition. Adv Nutr. 2014;5(5):563-5.
- Doherty S. History of evidence-based medicine. Oranges, chloride of lime and leeches: Barriers to teaching old dogs new tricks. EMA - Emergency Medicine Australasia. 2005;17(4):314-21.
- Gardner JK, Rall LC, Peterson C. Lack of Multidisciplinary Collaboration Is a Barrier to Outcomes Research. Journal of the American Dietetic Association. 2002;102(1):65-71.
- Hise ME, Kattelmann K, Parkhurst M. Evidence-based clinical practice: dispelling the myths. Nutrition in Clinical Practice. 2005;20(3):294-302.
- Ioannidis JP. Implausible results in human nutrition research. BMJ. 2013;347:f6698.
- Ioannidis JP. We need more randomized trials in nutrition-preferably large, long-term, and with negative results. The American Journal of Clinical Nutrition. 2016;103(6):1385-6.
- Marx W, Kiss N, McCarthy A, Isenring E. The attitudes, beliefs, and behaviors of healthcare professionals regarding dietary supplements. Supportive Care in Cancer. 2015;Conference: 2015 International MASCC/ISOO Symposium: Supportive Care in Cancer Copenhagen Denmark.
- Oakley Jr GP, Johnston Jr RB. Balancing benefits and harms in public health prevention programmes mandated by governments. British Medical Journal. 2004;329(7456):41-3.
- Otten JJ, Dodson EA, Fleischhacker S, Siddiqi S, Quinn EL. Getting Research to the Policy Table: a Qualitative Study With Public Health Researchers on Engaging With Policy Makers. Preventing Chronic Disease. 2015;12.
- Thomas DE, Kukuruzovic R, Martino B, Chauhan SS, Elliott EJ. Knowledge and Use of Evidence-Based Nutrition: a Survey of Paediatric Dietitians. Journal of Human Nutrition and Dietetics. 2003;16(5):315-22.
- Varma J, Rodriguez R, Mansi IA. Reasoning and evidence-based medicine: common pitfalls. Southern Medical Journal. 2012;105(3):167-72.
